# Excess mortality in adults from Sao Paulo during the COVID-19 pandemic in 2020: analyses of all-cause and noncommunicable diseases mortality

**DOI:** 10.1038/s41598-023-50388-7

**Published:** 2023-12-27

**Authors:** Bruna de Souza Resende, Rayara Mozer Dias, Gerson Ferrari, Leandro F. M. Rezende

**Affiliations:** 1 Department of Preventive Medicine, Escola Paulista de Medicina, Universidade Federal de São Paulo, Sao Paulo, Brazil; 2 Department of Epidemiology, Instituto de Medicina Social Hesio Cordeiro, Universidade Do Estado Do Rio de Janeiro, Rio de Janeiro, Brazil; 3 Universidad de Santiago de Chile (USACH), Escuela de Ciencias de La Actividad Física, El Deporte y La Salud, Santiago, Chile; 4 Faculty of Health Sciences, Universidad Autónoma de Chile, Providencia, Chile

**Keywords:** Diseases, Health care, Risk factors

## Abstract

In this study, we estimated the excess mortality from all-causes of death and noncommunicable diseases (NCDs) in adults living in the state of São Paulo during the COVID-19 pandemic in 2020. Number of deaths were retrieved from the Mortality Information System before (2017–2019) and during (2020) the COVID-19 pandemic, considering the following underlying causes of death: Neoplasms; Diabetes Mellitus; Circulatory System Diseases, and Respiratory System Diseases. Standardized Mortality Ratio (SMR) were calculated by dividing the mortality rates in 2020 by average mortality rates in 2017–2019*,* according to sex, age group, geographic location (state, capital, and Regional Health Departments). In 2020, occurred 341,704 deaths in the state of São Paulo *vs* 290,679 deaths in 2017–2019, representing an 18% increase in all-cause mortality (SMR 1.18) or 51,025 excess deaths during the first year of COVID-19 pandemic. The excess mortality was higher in men (186,741 deaths in 2020 vs 156,371 deaths in 2017–2019; SMR 1.18; 30,370 excess deaths) compared to women (154,963 deaths in 2020 vs 134,308 deaths in 2017–2019; SMR 1.15; 20,655 excess deaths). Regarding NCDs mortality, we observed a reduction in cancer mortality (SMR 0.98; −1,354 deaths), diseases of the circulatory system (SMR 0.95; −4,277 deaths), and respiratory system (SMR 0.88; −1,945). We found a 26% increase in Diabetes Mellitus mortality (SMR 1.26; 2885 deaths) during the pandemic year. Our findings corroborate the need to create and strengthen policies aimed at the prevention and control of NCDs, in order to mitigate the impact of future infectious disease pandemics.

## Introduction

Noncommunicable Diseases (NCDs) constitute the largest burden of morbidity and mortality in the world, accounting for, approximately, 70% of all annual deaths. In Brazil, NCDs account for 74% or 975,400 deaths per year. Cardiovascular diseases are the leading causes of NCDs deaths, followed by cancer, respiratory diseases and diabetes mellitus^1,2^. In addition to NCDs, the COVID-19 pandemic presented itself as one of the greatest health challenges on a global scale of the twenty-first century^3^, particularly considering that people living with NCDs had a higher risk of developing severe COVID-19 conditions, such as severe acute respiratory syndrome (SARS)^4^. Because of the synergism of these two epidemics to produce morbidity and mortality, some have argued that a syndemic approach is needed to better understand and protect the health of our communities^5,6^.

Social distancing and social restriction measures were adopted to reduce the spread of the coronavirus, but these preventive measures may have caused adverse consequences, such as changes in people’s lifestyles, as observed in Brazil^7,8^ and other countries^9,10^. In addition, it may have caused a reduction and/or temporary suspension of health procedures, actions, and services, as well as an increased demand for hospital care due to COVID-19. The reallocation of health resources to cope with COVID-19 presented an additional challenge in maintaining the necessary health care for people living with NCDs^11^. In May 2020, the World Health Organization (WHO) conducted a survey evaluating the provision of services for NCDs during the COVID-19 pandemic, identifying that 75% of participating countries reported interruptions in NCDs services^12^. In the Americas, the main reasons for the reduction and/or interruption of NCDs services included the cancellation of elective care services, healthcare teams being relocated to respond to COVID-19 and the patients’ non-attendance to in-person appointments^13^. In the state of São Paulo, the Resolution SS-28 of March 17, 2020 foresee the possibility of canceling and suspending various activities, such as outpatient consultations, diagnostic tests, surgical procedures, and elective surgeries. The document recommended the maintenance of these activities only when the benefits of prompt completion outweigh the risks associated with the COVID-19, especially for at-risk patients (adults over 60 years of age, patients with “comorbidities,” and immunosuppressed)^14^.

The overlap of COVID-19 and NCDs in space and time constituted an additional challenge within the scope of health services and the Brazilian Unified Health System^15^. Considering millions of people living with NCDs, deaths related to NCDs complications in patients not infected by the new coronavirus could eventually become more severe and significant than deaths directly affected by SARS-CoV-2 infection^16^. Epidemiological studies quantifying the potential the impact of COVID-19 on morbidity and mortality may provide important information to guide the planning of actions and public policies^17^. Excess all-cause and NCDs mortality have been used as a proxy of the overall impact of the COVID-19 pandemic on population health from different countries and socioeconomic groups^18–20^.

In this study, we estimated the excess all-cause and NCDs mortality in adults living in the state of São Paulo during the COVID-19 pandemic in 2020. Excess deaths were estimated using all deaths in 2020 by NCDs (cancer, diabetes mellitus, diseases of the circulatory system, and diseases of the respiratory system), sex, age group, geographic location (state, capital, and regional health departments) and month of the year.

## Methods

Epidemiological study carried out based on secondary data including all deaths in adults (≥ 18 years) residing in the state of São Paulo from the months of January to December, of the years 2017–2019 and 2020. Data on mortality were retrieved from the Mortality Information System of the state of São Paulo, obtained via the TabNet system of the State Department of Health of São Paulo. We conducted a comparative analysis of all-cause and NCDs deaths in the state of São Paulo before (2017–2019) and during (2020) the COVID-19 pandemic. The underlying causes of death were collected according to the International Classification of Diseases 10th revision (ICD-10) and the goals of the Brazilian Strategic Action Plan to Cope with NCDs in Brazil 2021–2030^21^: all-causes of death, cancer (C00-C97), diabetes mellitus (E10-E14), diseases of the circulatory system (I00-I99), and diseases of the respiratory system (J30-J98). Of note, São Paulo has one of the best quality Mortality Information System in Brazil, with less than 5% of unknow/not classified underlying causes of death^21^. The study was approved by the Research Ethics Committee of the Federal University of São Paulo (EPM/UNIFESP), approved under the CAAE No. 4,637,943 of April 8, 2021. All research methods were performed in accordance with relevant guidelines/regulations. The need for informed consent was waived by the approving Ethical Committee of the Federal University of Sao Paulo (EPM/UNIFESP) due to retrospective nature of the study. .

Data analysis was carried out from the construction of three databases on the deaths of residents for the state of São Paulo, for the capital, and for Regional Health Departments (*Departamentos Regionais de* Saúde—DRS). The administrative division of the São Paulo State Department of Health (SES/SP) takes place through the DRS^22^ that divided the state into 17 departments (I. Grande São Paulo, II. Araçatuba, III. Araraquara, IV. Baixada Santista, V. Barretos, VI. Bauru, VII. Campinas, VIII. Franca, IX. Marília, X. Piracicaba, XI. Presidente Prudente, XII. Registro, XIII. Ribeirão Preto, XIV. São João da Boa Vista, XV. São José do Rio Preto, XVI. Sorocaba, and XVII. Taubaté) responsible for coordinating regional activities^14^. Deaths in the DRS were analyzed annually in the previous period (2017–2019) and during the pandemic (2020), in the seventeen DRS of the state, analyzing all-causes of death and NCDs deaths by sex and age groups (15–19 years, 20–29 years, 30–39 years, 40–49 years, 50–59 years, 60–69 years, 70–79 years, 80 and over). Deaths in the state and capital were analyzed monthly in the pre-pandemic period (2017–2019) and in the pandemic period (2020) for all-causes and NCDs deaths by sex and age groups.

Standardized Mortality Ratio (SMR) was calculated by dividing the mortality rates in 2020 by the number of deaths expected for that year, the latter being calculated from the average number of deaths and population size in the triennium that preceded the COVID-19 pandemic (2017–2019). Excess deaths were calculated by subtracting the observed number of deaths minus the expected number of deaths in 2020, being the latter calculated as the average mortality rates in 2017–2019 times the population projected for 2020 in the state. Population data by year and by municipality were obtained from population projections available on the DATASUS TabNet (Department of Informatics of SUS). The mortality rate per 100,000 inhabitants was calculated according to the underlying cause of death by ICD-10, considering in the analyses the sociodemographic characteristics of the population of the state of São Paulo.

Data analysis was performed using the Excel 2016 software and *Qgis 3.28* Software. The classes of the SMR intervals presented in the maps were defined using the mode of natural breaks, also known as Jenks Criterion, which considered the automatic distribution of the values of each interval seeking to minimize the variance within each class, increasing the variance between the different classes^23^.

## Results

**Figure 1:**
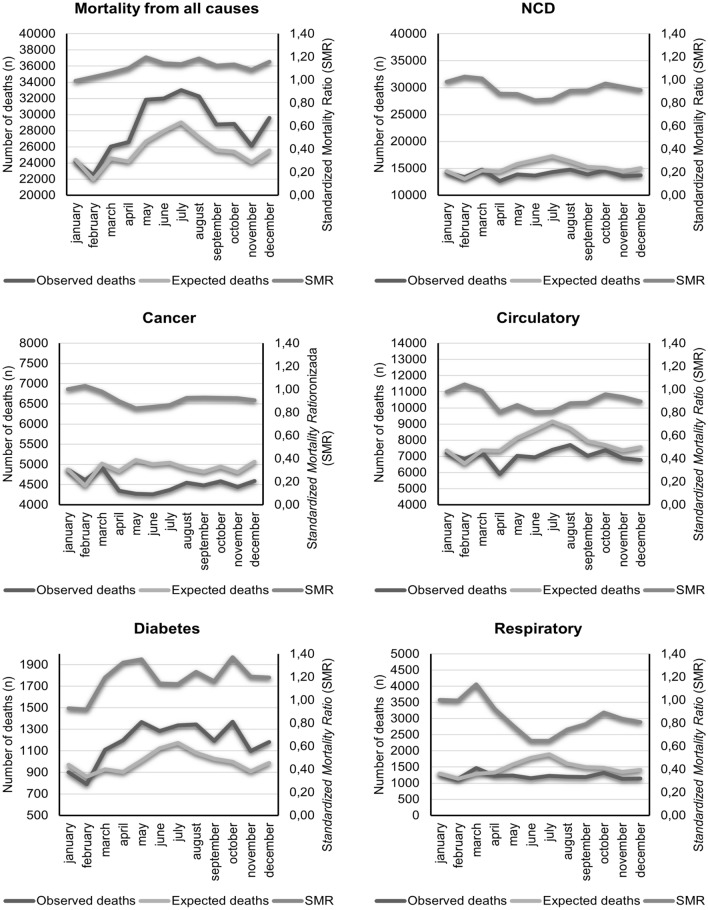
Standardized mortality ratio, number of deaths observed and expected in the state of São Paulo in 2020. São Paulo, 2020.

In 2020, occurred 341,704 deaths in the state of São Paulo *vs* 290,679 deaths in 2017–2019, representing an 18% increased all-cause mortality (SMR 1.18) or 51,025 excess deaths during the first year of COVID-19 pandemic. The excess mortality was higher in men (186,741 deaths in 2020 vs 156,371 in 2017–2019; SMR 1.18; 30,370 excess deaths) compared to women (154,963 deaths in 2020 vs 134,308 in 2017–2019; SMR 1.15; 20,655 excess deaths). Regarding NCDs mortality, we observed a reduction in the number of deaths from cancer (SMR 0.98; −1,354 deaths), diseases of the circulatory system (SMR 0.95%; −4277 deaths), and respiratory system (SMR 0.88; −1945), and an increase in the number of deaths from Diabetes Mellitus (SMR 1.26; 2885 deaths) during the pandemic year. Overall, the number of NCDs deaths in men decreased from 89,914 deaths in 2017–2019 to 87,194 deaths in 2020, whereas in women these figures were 82,308 deaths in 2017–2019 to 80,337deaths in 2020 (Table 1). Figure 1 displays the monthly SMR and excess deaths from all-causes and NCDs deaths in the State of Sao Paulo.

**Table 1:** Standardized Mortality Ratio, number of deaths and excess deaths from all-cause and noncommunicable diseases deaths in the State of São Paulo and in the capital according to sex. São Paulo, 2017–2019 and 2020.

Location/Mortality	Men			Women			Total					
	Deaths			Deaths			Deaths					
	Annual Average	2020	SMR	Excess deaths in 2020*	Annual Average	2020	SMR	Excess deaths in 2020*	Annual Average	2020	SMR	Excess deaths in 2020*
	2017–2019				2017–2019				2017–2019			
State—SP												
All-cause	156,371	186,741	1.19	30,370	134,308	154,963	1.15	20,655	290,679	341,704	1.18	51,025
NCDs	89,914	87,194	0.97	−2,720	82,308	80,337	0.98	−1,971	172,222	167,531	0.97	− 4,691
Cancer	29,208	28,085	0.96	−1,123	26,428	26,197	0.99	−231	55,636	54,282	0.98	− 1,354
Diabetes Mellitus	5,265	6,797	1.29	1,532	6,026	7,379	1.22	1,353	11,291	14,176	1.26	2,885
Circulatory system diseases	46,545	44,278	0.95	−2,267	42,089	40,079	0.95	−2,010	88,634	84,357	0.95	− 4,277
Respiratory diseases	8,896	8,034	0.9	−862	7,765	6,682	0.86	−1,083	16,661	14,716	0.88	− 1,945
Capital—SP												
All-cause	38,354	47,398	1.24	9.044	36,538	42,799	1.17	6,261	74,892	90,197	1.20	6,270
NCDs	23,260	19,983	0.86	−3277	23,392	20,718	0.89	−2674	46,652	40,701	0.87	− 5,951
Cancer	7,587	6,579	0.87	−1008	7,874	7,231	0.92	−643	15,461	13,810	0.89	− 1,651
Diabetes	1,266	1,347	1.06	81	1,376	1,437	1.04	61	2,642	2,784	1.05	142
Circulatory system diseases	12,336	10,572	0.86	−1764	12,156	10,664	0.88	−1,492	24,492	21,236	0.87	− 3,256
Respiratory diseases	2,070	1,485	0.72	−585	1,986	1,386	0.7	−600	4,056	2,871	0.71	− 1,185

In the city of São Paulo, the average annual deaths in 2017–2019 were 38,354 in men and 36,538 in women. In 2020, 47,398 deaths occurred in men and 42,799 in women, representing an increase of 23.6% in the number of deaths in men and 17.1% in women (Table 1). Regarding the NCDs deaths, we observed a reduction in the number of deaths from cancer (−10.7%), diseases of the circulatory system (−13.3%), and of respiratory system (−29.2%). On the other hand, we observed an increase in the number of deaths from Diabetes Mellitus (5.4%), with an excess of 81 deaths in men and 61 in women (SMR of 1.06 and 1.04, respectively).

Regarding the age groups, Table 2 shows that the lowest SMR occurred in the age group of 15 to 19 years, with 0.92 for men in the state and in the municipality, and 1.01 in the state and 1.12 in the municipality for women. In women, SMR in the age group of ≥ 80 years was the second lowest, both in the state and in the municipality (1.11 and 1.17, respectively), being only higher than the age group of 15 to 19 years. In men, the age group ≥ 80 years presented SMR of 1.19 in the state and 1.22 in the municipality.

**Table 2:** Standardized mortality ratio, number of deaths, and excess deaths from all-cause and NCDs deaths in the state of São Paulo and in the capital according to age-group and sex. São Paulo, 2017–2019 and 2020.

Location	Men				Women				Total			
	Deaths				Deaths				Deaths			
	Average	2020	SMR	Excess deaths in 2020*	Average	2020	SMR	Excess deaths in 2020*	Average	2020	SMR	Excess deaths in 2020*
Age group (years)	2017–2019				2017–2019				2017–2019			
State—SP												
15 to 19	1741	1,599	0.92	−142	584	588	1.01	4	2325	2,187	0.94	−138
20 to 29	5394	5,812	1.08	418	1798	2,106	1.17	308	7192	7,918	1.10	726
30 to 39	7459	8,764	1.18	1,305	3474	3,980	1.15	506	10,933	12,744	1.17	1,811
40 to 49	12,109	14,593	1.21	2,484	6690	8,025	1.2	1,335	18,799	22,618	1.20	3,819
50 to 59	22,887	26,743	1.17	3,856	13,264	15,198	1.15	1,934	36,151	41,941	1.16	5,790
60 to 69	33,558	41,379	1.23	7,821	22,432	27,252	1.21	4,820	55,990	68,631	1.23	12,641
70 to 79	34,872	42,394	1.22	7,522	29,739	35,026	1.18	5,287	64,611	77,420	1.20	12,809
80 and more	38,352	45,457	1.19	7,105	56,327	62,788	1.11	6,461	94,679	108,245	1.14	13,566
Capital—SP												
15 to 19	546	503	0.92	-43	157	176	1.12	19	703	679	0.97	-24
20 to 29	1481	1,729	1.17	248	510	648	1.27	138	1991	2,377	1.19	386
30 to 39	1853	2,322	1.25	469	865	1,059	1.22	194	2718	3,381	1.24	663
40 to 49	2960	3,683	1.24	723	1718	2,056	1.2	338	4678	5,739	1.23	1,061
50 to 59	5507	6,687	1.21	1,180	3,350	3,879	1.16	529	8857	10,566	1.19	1,709
60 to 69	8064	10,228	1.27	2,164	5710	7,052	1.24	1,342	13,774	17,280	1.25	3,506
70 to 79	8168	10,302	1.26	2,134	7666	9,171	1.2	1,505	15,834	19,473	1.23	3,639
80 and more	9,776	11,944	1.22	2,168	16,561	18,758	1.13	2,197	26,337	30,702	1.17	4,365

Abbreviation: Standardized Mortality Ratio (SMR): ratio of mortality (age-adjusted) observed in 2020 by the mortality observed in the period 2017–2019. *Excess deaths: difference in the number of deaths observed in 2020 by the expected deaths in 2020 if the mortality rate (adjusted for age) for the period 2017–2019 were maintained.

**Figure 2:**
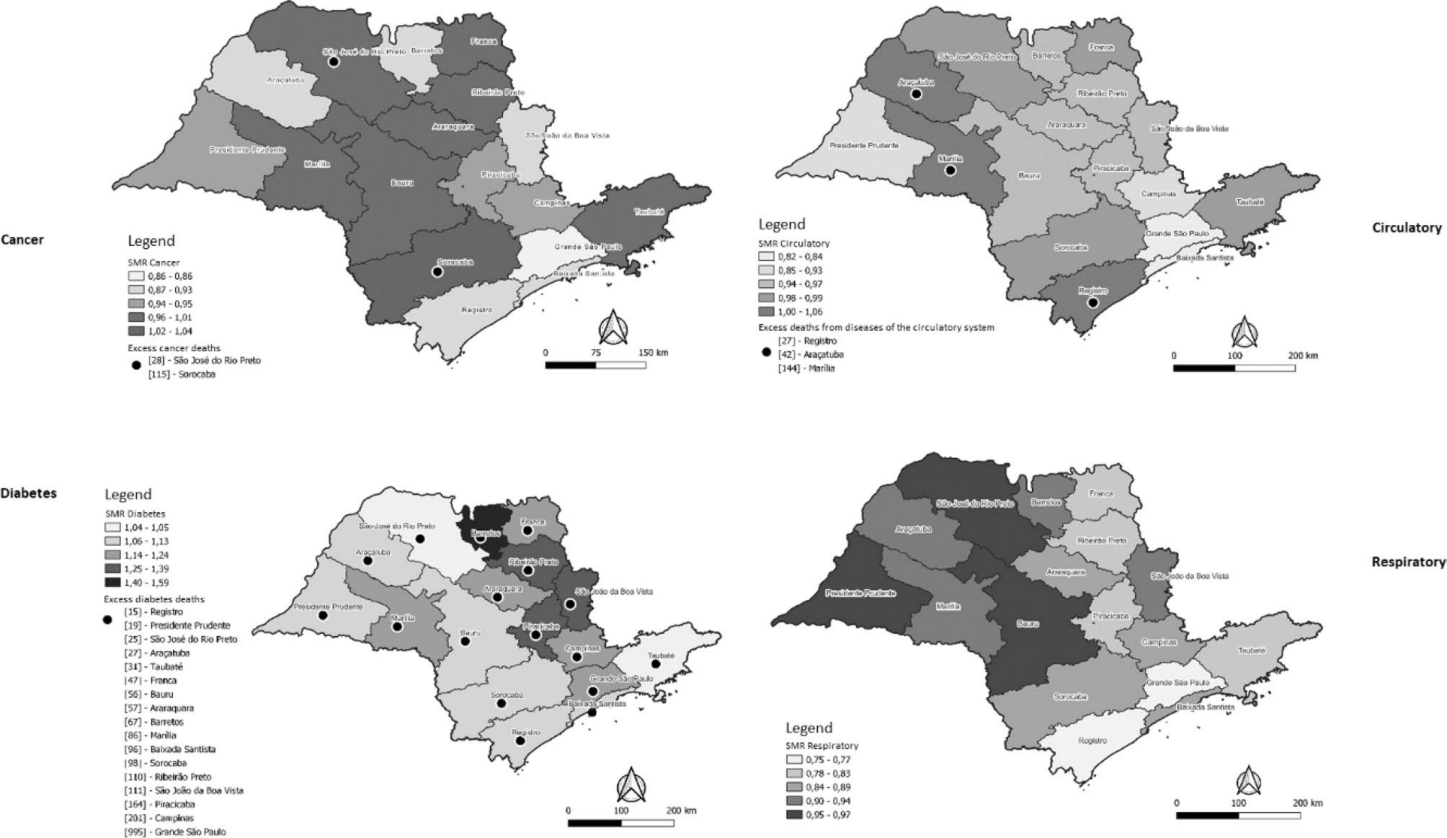
Standardized Mortality Ratio (SMR) by noncommunicable diseases and by Regional Health Departments of the state of São Paulo. São Paulo, 2020. The maps were generated in Qgis 3.28 Software—https://www.qgis.org/pt_BR/site/index.html.

Figure 2 represents the SMR and excess deaths for each grouping of NCDs in the Regional Health Departments of the state of São Paulo. Two regions had SMR higher than 1.00 for cancer mortality, São José do Rio Preto (1.01) and Sorocaba (1.04), presenting excess of 28 and 115 deaths, respectively. In regard to mortality due to diabetes mellitus, all regionals had SMR greater than 1.00, with the Regional of Barretos (SMR 1.59) having the highest excess of deaths (67 deaths), followed by the Regional of Piracicaba (SMR 1.39), with an excess of 164 deaths, and the Regional of São João da Boa Vista (SMR 1.32), which had an excess of 111 deaths. Regarding mortality from diseases of the circulatory system, three regions had SMR greater than 1.00: Marília (1.06), Registro (1.04), and Aracatuba (1.03), with excess deaths of 144, 27, and 42, respectively. None of the regions had SMR for diseases of the respiratory system equal to or greater than 1.00; no excess deaths were identified within this group of NCDs in the state.

## Discussion

Our study estimated an increase in overall mortality during the first year of the COVID-19 pandemic (2020) compared with the previous three-year period (2017–2019) in adults living in the state of São Paulo. In the same period, there was a reduction in mortality from cancer, diseases of the circulatory and respiratory system, and an increase in the number of deaths from diabetes mellitus. Similar results were observed for the city of São Paulo and the 17 DRSs, particularly those placed in the northern region of Sao Paulo. Although we have not explored the predictors of regional differences, future studies may be important to better understand the explanation for this pattern.

Several studies, both at the national and international levels, have estimated excess mortality from all-cause and cause-specific mortality since the beginning of the COVID-19 pandemic. A study with 22 countries worldwide, including Brazil, indicated excess all-cause mortality based on the five years prior to the pandemic. It was identified that Brazil (SMR: 1.13; excess of 120,220 deaths), France (SMR: 1.03; excess of 13,700 deaths), Italy (SMR: 1.03; excess of 11,103 deaths), Spain (SMR: 1.17; excess of 48,643 deaths), the United Kingdom (England [SMR: 1.15; excess of 53,869 deaths], Wales [SMR: 1.15; excess of 53,869 deaths], Northern Ireland [SMR: 1.08; excess of 825 deaths], and Scotland [SMR: 1.10; excess of 3911 deaths]), and the United States (SMR:1.2; excess of 231,327 deaths) had excess mortality from all-cause, while Australia (SMR: 0.97; −2083 deaths), Denmark (SMR: 0.96; −1339 deaths), and Georgia (SMR: 0.97; −839 deaths) showed a decrease in the number of deaths. These findings were correlated with geographic location and seasonality, as well as governments’ swiftness in applying preventive measures to reduce the spread of the COVID-19^24^. Another modelling study in Nordic countries (Denmark, Finland, Iceland, Norway, and Sweden) suggested that 15,000 to 20,000 excess deaths occurred during 2020–2021^25^.

Studies analyzing the excess mortality have gained temporal continuity, with the extension of the analyses of this pandemic effect also for the years 2021 and 2022. A study showed that the US continued with excess mortality from all-causes of death compared to similar countries, such as Germany, the Netherlands, Austria during the year 2021 and early 2022; a difference that is responsible for 150,000 to 470,000 deaths, which was mitigated in the 10 states with the highest vaccination coverage in the country^26^. While some national and international studies associate excess all-cause mortality with COVID-19 mortality^27–29^, others call attention to a low percentage of excess deaths confirmed by COVID-19, suggesting that many deaths from the disease may not have been documented, as was the case in Ecuador for the year 2020, in which only 20% of excess deaths were confirmed as being caused by the new coronavirus^30^.

Regarding the underlying cause of deaths, our study identified a reduction in the number of deaths from cancer (−2.0%, SMR = 0.96 in women and 0.99 in men) and diseases of the circulatory system (−4.8%, SMR = 0.95) in the state of São Paulo. In Brazil, a previous study has also estimated the reduction in the number of NCDs deaths during the pandemic period. Their study showed that the number of cancer deaths in Brazil in 2020 was 10% lower than expected, based on the mortality profile in the same period in 2019 for the country (SMR = 0.90; 95%CI 0.90–0.91). Similar findings were observed for cardiovascular diseases (SMR = 0.91; 95%CI 0.91–0.920). A possible explanation for these findings is the overlap of COVID-19 as a competitive cause of death, which may have resulted in migration of cause of death. In fact, previous studies have reported an increased number of reports of cancer and cardiovascular diseases as “comorbidities” in death certificates^31^. Therefore, prevalent cases of cancer and diseases of the circulatory system, which would have a higher risk of death from these diseases, may have had their deaths anticipated due to COVID-19^31,32^.

Modelling studies have estimated the future impact of the COVID-19 pandemic on mortality due to delay in cancer diagnosis. Research in countries such as the United Kingdom, France, Australia, and India points to estimates of additional deaths/excess mortality from different types of cancer in the coming post-pandemic years^33–35^. In France, an excess of 1,000 to 6,000 deaths was estimated in the coming years^33^, while in the United Kingdom the proportion of additional cancer deaths was estimated in the coming years, being identified between 15.3 and 16.6% of additional deaths due to colorectal cancer, 7.9% to 9.6% due to breast cancer, 5.8% to 6.0% due to esophageal cancer, and 4.8% to 5.3% due to lung cancer^35^. In Australia, the impact of additional deaths caused by changes in the histological stage of cancer at the beginning of treatment was predicted for breast, lung, colorectal and melanoma cancers, whose evolutionary stages may be more advanced, which will also cause problems related to higher health costs^34^.

A Brazilian study pointed out a reduction in the number of hospital deaths due to diseases of the circulatory system in 2020, represented by 1,495 fewer deaths than expected. However, this same study pointed out that the Midwest region showed a 15.1% increase in the number of deaths from diseases of the circulatory system, and that ten Federation Units also had a higher number of deaths in 2020, not including the state of São Paulo^36^, a finding that is in line with our findings. However, it is worth mentioning that one of the impact points of COVID-19 may be related to changes in the place of occurrence of deaths, including the increase in home deaths due to limited access to services from the overload of health services, and isolation and social restriction measures that hindered the early detection of cardiovascular symptoms and immediate treatment^18^.

Regarding mortality from diabetes mellitus, this study indicated an increase in the number of deaths (25.6%) with an excess of 1,532 deaths in men and 1,353 deaths in women in 2020 (SMR of 1.29 and 1.22, respectively). These findings are in line with an American study that pointed out that an increase in diabetes mortality was observed in most American states, associated with the pandemic in general (relative change in mortality rates 1.19 [95%CI 1.13, 1.25]) and in 24 states, with the highest proportion of relative change in mortality rates in Mississippi (1.46 [95%CI 1.23, 1.72]), followed by New Jersey (1.44 [95%CI 1.08, 1.91]) suggesting indirect impacts of the COVID-19 pandemic on the comprehensive health care of people living with diabetes mellitus^37^. Among the possible explanations for the increase in mortality from diabetes mellitus during the COVID-19 pandemic are: hesitation of patients with severe symptoms of the disease to receive hospital medical services during the most severe phases of the COVID-19 pandemic in 2020, with the concern of in-hospital infection by the new coronavirus; occurrence of early hospital discharge of patients with diabetes mellitus, since the pandemic overwhelmed health services, especially hospital care^37^; restrictions on outpatient care for diabetes and possible delays in emergency services^38^ and a possible reduction in the quality of management for patients with diabetes mellitus in the context of the pandemic, capable of increasing mortality of the disease in the absence of timely treatment^37–39^.

A Brazilian study carried out in 171 municipalities in the state of São Paulo identified that most municipalities (89.6%) defined a set of health services to be maintained during the pandemic, with 95.7% reporting discontinuities in health care for people living with NCDs. Among the services with discontinuity (total interruption and partial interruption) were elective surgeries (54.1% and 38.1%), rehabilitation (10.0% and 62.1%), diagnosis/treatment of NCDs (1.0% and 42.1%), diagnosis/treatment of cancer (partial interruption 15.9%), and palliative care (4.4% and 22.6%)^40^. Of note, diabetes mellitus requires the use of medications, continuous monitoring, and comprehensive care at different levels to prevent complications that can increase mortality from the disease. Therefore, the need for isolation, the difficulty of using transport and the risk of people living with diabetes mellitus developing the most severe form of COVID-19 may have created the false impression that avoiding COVID-19 was more urgent than controlling the complications resulting from the decompensation of diabetes^41^.

Regarding the findings on the reduction of mortality from diseases of the respiratory and circulatory system in São Paulo, they corroborate with national research, which observed reductions in deaths from these two groups of diseases in Brazil in 2020^42^. The fact that there were no excess deaths due to diseases of the respiratory system in São Paulo may corroborate the idea that many deaths due to respiratory causes were reported as COVID-19 deaths, which started to compose a new special chapter in the Mortality Information System.

This study has some limitations. During data collection, a small divergence (< 2%) was observed between the number of deaths in the state and the sum of the number of deaths obtained across DRS, due to missing values in the “municipality of residence” in the state of São Paulo. We have considered the average of deaths and population size between 2017–2018 to estimate the expected number of deaths in 2020, had the Covid-19 not occurred. Although similar methods have been applied elsewhere^43^, we recognize the expected number of deaths could have been different, considering temporal trends in cause-specific NCD mortality. However, our findings may provide a comprehensive and useful information on excess mortality in the state of São Paulo during the COVID-19 pandemic. Of note, São Paulo has one of the highest quality mortality information systems in the country, with less than 5% of unknow/not classified underlying causes of death.

## Conclusions

In conclusion, we estimated a 18% increased all-cause mortality (SMR 1.18) or 51,025 excess deaths during the first year of COVID-19 pandemic compared to 2017–2019. In the same period, however, there was a reduction in mortality from cancer, circulatory system disease, respiratory disease, and increased mortality from diabetes mellitus. Our findings reinforce the importance of formulating and implementing public policies that guarantee the prevention and control of NCDs. These measures are imperative to mitigate the detrimental health effect on the health of people living with NCDs, particularly in situations of health emergency, such as the COVID-19 pandemic.

## Data Availability

The datasets used and/or analyzed during the current study available from the corresponding author on reasonable request.
